# The Anti-Inflammatory Effect of *Callicarpa nudiflora* Extract on *H. Pylori*-Infected GES-1 Cells through the Inhibition of ROS/NLRP3/Caspase-1/IL-1*β* Signaling Axis

**DOI:** 10.1155/2022/5469236

**Published:** 2022-07-15

**Authors:** Lili Li, Bo Bao, Xingxing Chai, Xiaoyu Chen, Xiaohua Su, Shixiu Feng, Xiaohui Zhu

**Affiliations:** ^1^Laboratory Animal Centre, Guangdong Medical University, Dongguan 523808, China; ^2^Department of Pathophysiology, School of Basic Medicine Sciences, Guangdong Medical University, Zhanjiang 524023, China; ^3^Key Laboratory of South Subtropical Plant Diversity, Shenzhen and Chinese Academy of Sciences, Fairy Lake Botanical Garden, Shenzhen 518004, China; ^4^College of Pharmacy, Shenzhen Technology University, Shenzhen 518118, China

## Abstract

*Helicobacter pylori* (*H. pylori*) is the main pathogenic factor of gastric cancer, chronic gastritis, and other gastric diseases. It has been found that *Callicarpa nudiflora* (CN) as an air-dried leaf extract has a broad-spectrum antibacterial effect. This study aims to examine the effect of CN on *H. pylori*-infected GES-1 cells *in vitro* and elucidate its underlying mechanism by extracting active ingredients from air-dried leaves. GES-1 cells were cocultured with HPSS1 at MOI = 100 : 1 and treated with different concentrations of CN (100 and 200 *μ*g/ml). Results showed that CN can significantly reduce cellular LDH leakage and attenuate *H. pylori*-induced cell apoptosis and ROS production in GSE-1 cells, so as to protect gastric epithelial cells from damage by *H. pylori*. CN can also inhibit the secretion of inflammatory factors, such as TNF-*α*, IL-1*β*, IL-6, and IL-8. After CN treatment, the expression levels of active caspase-1, PYCARD, and NLRP3 were remarkably decreased in the treatment groups compared with the model group. To sum up, CN is highly protective against *H. pylori*-induced cell damage and apoptosis; CN can depress NLRP3 inflammasome activation and ROS production via the ROS/NLRP3/caspase-1/IL-1*β* signaling axis to suppress *H. pylori*-triggered inflammatory response and pyroptosis.

## 1. Introduction

*Helicobacter pylori* (*H. pylori*) is a Gram-negative spiral bacterium and infects almost half of the world's population [1]. In most cases, patients infected with *H. pylori* have no symptoms. Only ∼30% of the infected patients may progress to clinical symptoms [2]. *H. pylori* colonizes human gastric epithelial cells to bring about neutrophil infiltration and gastric mucosal layer edema, leading to chronic gastritis, atrophic gastritis, and even gastric cancer [1–3]. *H. pylori* is not only the cause of more than 70% of gastritis but also the main carcinogen of gastric cancer by WHO [4]. It was first isolated and described in 1983 by Barry Warren and Robin Marshall, who discovered the bacterium *H. pylori* and its role in gastritis and peptic ulcer diseases to win the 2005 Nobel Prize in physiology or medicine [5]. *H. pylori* can express a variety of pathogenic virulent factors, one of which is the cytotoxin-associated gene A (CagA). CagA can trigger the expression of proinflammatory cytokines by activating the expression of nuclear factor (NF)-*κ*B of gastric epithelial cells [6]. *H. pylori* can also induce the release of proinflammatory factors and the associated oxidative damage. For instance, the proinflammatory factor IL-8 can activate and aggregate neutrophils, thereby inducing reactive oxygen species (ROS) production [7]. Excessive ROS can activate the NLRP3 inflammasome to trigger the cleavage of pro-IL-1*β* and transform it into activated IL-1*β* [8, 9].

Currently, according to the international and domestic consensus, the preferred regimen for eradicating *H. pylori* infection is quadruple therapy, mainly including proton pump inhibitors, bismuth agents, and two antibiotics. However, this treatment has some shortcomings such as drug resistance, gastrointestinal flora imbalance, and indigestion [10, 11]. Therefore, it is urgent to develop effective, low toxicity drugs for the therapy of *H. pylori* infection. Chinese herbal medicine (CHM) has been widely used to eliminate infections due to its multitarget effects and fewer side effects [12, 13]. Some herbs have shown good anti-infection potential in *H. pylori*-associated gastritis [14–16].

*Callicarpa nudiflora* Hook. Ex Arn. belongs to the genus *Callicarpas linn. o*f the family Verbenaceae, whose dry leaves are used as a traditional Chinese herbal medicine. It is mainly distributed in Guangdong, Hainan, and Guangxi provinces of China [17]. The main chemical constituents include phenylpropanoids, flavonoids, triterpenoids, diterpenoids, cycloene ether terpenoids, and phenolic acids as well as their glycosides and sterols [18]. According to “The Supplement of Compendium of Materia Medica” and Chinese Pharmacopoeia 2015, it can not only relieve poisonous toxin, gangrene, laryngeal paralysis, poisonous swelling, pain, wind-evil, and other symptoms but also treat respiratory infection, hepatitis, and bleeding [19, 20]. Because of its broad-spectrum antibacterial effect, *Callicarpa nudiflora* Hook. Ex Arn. exerts different degrees of inhibition on *Staphylococcus aureus*, *Salmonella typhi*, *Pneumococcus*, *Pseudomonas aeruginosa*, *Escherichia coli*, and *Shigella*. Previously, *Callicarpa nudiflora* (CN) extract was prepared from the dry leaves of *Callicarpa nudiflora* Hook. Ex Arn. to analyze its main chemical constituents and evaluate the corresponding pharmacological activities [18].

This research mainly focuses on its pharmacological activity in inflammation, detects the therapeutic effect of CN on *H. pylori*-infected GES-1 cells, and further explores the molecular mechanism of how CN exerts its anti-inflammatory effect. Hopefully, this project will provide a candidate drug for the treatment of clinical *H. pylori*-associated gastritis.

## 2. Materials and Methods

### 2.1. Preparation of *Callicarpa Nudiflora*

The voucher specimen (SZG00048161) was deposited in the Plants Herbarium of Fairy Lake Botanical Garden (Shenzhen, China) (Supplementary Figure 1). *Callicarpa nudiflora* was prepared according to the China Pharmacopoeia 2015. The air-dried leaves of *Callicarpa nudiflora* were smashed into powder (about 80 meshes). One kilogram of powder was extracted with two liters of water, boiled to approximately 100°C and stored for 1 h. The extracted liquid was filtered and the residue was boiled with 1.5 liters of water for another 1 h. The collected decoction was mixed, followed by being freeze-dried (Eyela FDU-2110, Eyela Corp, Tokyo, Japan) to gain *Callicarpa nudiflora* powder (268 g). Next, the chemical constituents of *Callicarpa nudiflora* were identified by the UPLC-ESI-Q-TOF-MS (Supplementary Table 1). Additionally, the UPLC spectrum was used to analyze four main compounds of CN, with 33.26% of acteoside, 0.98% of forsythoside B, 0.42% of 5,4′-dihydroxy-3,7,3′-trimethoxyflavone, and 0.33% of luteolin (Supplementary Figure 2). *In vitro*, *Callicarpa nudiflora* was dissolved in DMSO that was used for control (the volume of DMSO *<* 0.5% in all experiments).

### 2.2. *H. pylori* Strain and Growth Condition

The HPSS1 was provided in a frozen state by the National Centers for Disease Control and maintained on the *Campylobacter* Karmaili Agar Base plates (CM0935B, OXOID, United Kingdom) and brain heart infusion broth (CM1136, OXOID, United Kingdom) supplemented with 5% sterile defibrated sheep blood (C035, Chun Du Biotechnology, China) under 85% N_2_, 10% CO_2_, and 5% O_2_ at 37°C for 48–72 h in the three-gas incubator (Heal Force, Shanghai, China).

### 2.3. Cell Culture and *H. pylori* Infection

GES-1 cells were purchased from iCell Bioscience Inc, Shanghai. Cells were propagated in RPMI-1640 media with 10% fetal bovine serum (10091–148, Gibco, United States) and 1% penicillin-streptomycin (15070–063, Gibco, United States) under 5% CO_2_ at 37°C. These cells were passaged at the time of reaching 80% confluence. Without penicillin-streptomycin, cells were then divided into four groups: control, model (*H. pylori*, HP) and CN 100, and 200 (100, and 200 *μ*g/ml CN with HP) groups. In the HP group, GES-1 cells were cocultured with *H. pylori* in a cell incubator at MOI = 100 : 1 for 24 h. In the CN groups, GES-1 cells were cocultured with *H. pylori* at MOI = 100 : 1 for 24 h, followed by treatment with different concentrations of CN for another 24 h.

### 2.4. Determination of MIC

The inhibitory effect of *Callicarpa nudiflora* extract (0.0025∼40 mg/ml) on HPSS1 was evaluated by the determination of the minimal inhibitory concentration (MIC). The CN solutions of different concentrations after double dilution were added onto a sterilized 96-well plate, and *H. pylori* culture in the logarithmic growth phase was scraped to prepare a bacterial suspension with a concentration of OD600 of 1. After a 1 : 1000 dilution in the medium, 50 *μ*l of bacterial suspension was added to each well. The control group consisted of CN with different concentrations without HPSS1; the positive control group consisted of normal cultured HPSS1 without CN; the plate was incubated in a three-gas incubator for 24 h, and the OD600 was measured.

### 2.5. Evaluation of GES-1 Cell Viability after Incubation with *H. pylori*

The cell viability of GES-1 cells exposed to *H. pylori* and CN was assessed by the CCK-8 assay and the percentage of LDH leakage according to kit instructions.

### 2.6. Apoptosis Detection

GES-1 cells with a good growth state in the logarithmic growth phase were inoculated into 6-well plates with 10^5^/well. 2 ml of culture medium was added to each well and cultured overnight in a 5% CO_2_ incubator at 37°C, followed by processing cells according to groups. The cell pellets were collected and washed in PBS, and resuspended cells with 500 *μ*l binding buffer, 5 *μ*l Annexin V-FITC, and 5 *μ*l PI were added and mixed. The reaction was removed from light for 5∼15 min at room temperature and detected by flow cytometry (Beckman coulter, cytoFLEX, United States).

### 2.7. Measurement of ROS

The cells treated above were collected and washed in PBS, followed by adding 1 ml DCFH-DA which was diluted in the serum-free medium to 10 M, incubated at 37°C for 20 min, and mixed once every 3 min. Cells were then washed twice with serum-free medium and resuspended with PBS to be tested by flow cytometry (Beckman coulter, cytoFLEX, United States).

### 2.8. ELISA Assay for TNF-*α*, IL-1*β*, IL-8, and IL-6

Supernatants from the culture of GES-1 cells were collected and centrifuged to remove cell debris. According to the manufacturer's instructions, the cytokine specific ELISA kit was used to determine the concentrations of IL-8 (RK00011, ABclonal, China), IL-1*β* (RK00001, ABclonal, China), IL-6 (E-EL-H0102c, Elabscience, China), and TNF-*α* (E-EL-H0109c, Elabscience, China) in the culture supernatants. All the assays were conducted in triplicate in three independent experiments (FlexStation® 3, Molecular Devices, United States).

### 2.9. Western Blot

Total protein was extracted with the RIPA lysis buffer containing phosphatase and protease inhibitors, and the concentration of protein was measured with the BCA Protein Assay Kit (P0010, Beyotime Institute of Biotechnology, China). SDS-PAGE was used to separate lysates, and then the proteins were transferred to PVDF membranes. At room temperature, the membranes were blocked with 5% nonfat milk for 2 h and incubated overnight with primary antibodies at 4°C. The signals were detected with ECL reagents after incubation with secondary antibodies. Antibodies used were: GAPDH (5174S, Cell Signaling Technology, United States), ASC (DF6304, Affinity Bioscience, United States), PARP1 (66520-1-AP, Proteintech, China), caspase-1 (SC-56036, Santa Cruz, United States), NLRP3 (DF7438, Affinity Bioscience, United States), and caspase-3 (9662S, Cell Signaling Technology, United States).

### 2.10. Statistical Analysis

GraphPad Prism 5.0 (GraphPad Software, La Jolla, CA) was used for data analysis. Different treatments were compared with unpaired *t*-tests using parametric tests. Data were indicated as the mean ± standard error, and differences were considered significant as follows: ^*∗*^*p* < 0.05; ^*∗∗*^*p* < 0.01; ^*∗∗∗*^*p* < 0.001; ^*∗∗∗∗*^*p* < 0.001; ns: not significant.

## 3. Results

### 3.1. CN Showed Anti-HPSS1 Activity in Vitro

**Figure 1:**
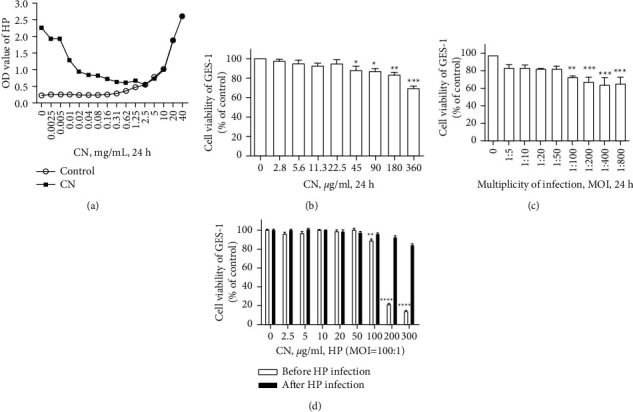
CN showed anti-HPSS1 activity in vitro. (a) The MIC value of CN on HPSS1. (b) Toxicity determination of CN to GES-1. (c) Determination of the proportion of GES-1 infected with HPSS1. (d) Comparison of the effects of CN before and after HPSS1 infection. ^*∗*^*p* < 0.05, ^*∗∗*^*p* < 0.01, ^*∗∗∗*^*p* < 0.001, ^*∗∗∗∗*^*p* < 0.001 compared with the control group.

Due to the broad-spectrum antibacterial effect of CN, the inhibitory effect of CN on HPSS1 was first detected, and the minimal inhibitory concentration test showed that the MIC value of CN on HPSS1 was 2.5 mg/ml, with no visible bacterial growth (Figure 1(a)). In order to figure out the toxic and negative effects of CN on GES-1 cells, the CCK-8 assay was conducted with increasing CN concentrations. Results showed that CN presented toxic effects at 45 *µ*g/ml, with a higher concentration causing stronger toxicity (Figure 1(b)). In order to further explore the role of CN in GES-1 infected by HPSS1, a complex number of infections were explored between HPSS1 and GES-1. According to the experimental results, the MOI of 100 was selected for the subsequent experimental study (Figure 1(c)). On this basis, the effect of CN was further studied before and after HPSS1 infection, showing that the effect of CN after HPSS1 infection was better (Figure 1(d)). Therefore, CN was chosen to be used for the treatment of HPSS1-infected GES-1 cells in the following studies.

### 3.2. CN Counteracted the Damage of *H. pylori* to GES-1 Cells

**Figure 2:**
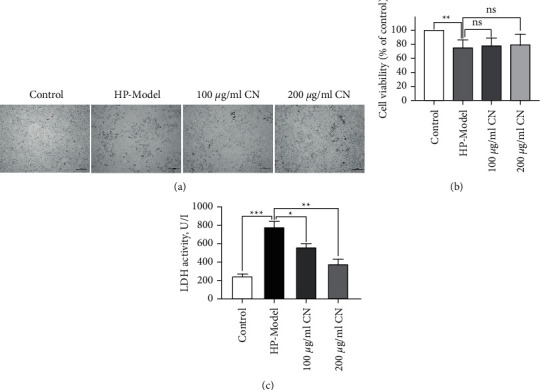
CN reduced the damage of *H. pylori* to GES-1. (a) Morphological characterization of GES-1. Scale bar, 100 *µ*m. (b) The effects of CN on cell viability of *H. pylori*-infected GES-1. (c) The effects of CN on the percentage of LDH leakage of *H. pylori*-infected GES-1. ^*∗*^*p* < 0.05, ^*∗∗*^*p* < 0.01, ^*∗∗∗*^*p* < 0.001, compared with the specified group.

The morphology, viability, and the percentage of LDH leakage of *H*. *pylori*-infected GES-1 cells were analyzed to investigate the effect of CN on cell physiology and survival after incubation with *H. pylori*. *In vitro*, GES-1 cells of the control group were in good condition, the cell morphology of the model group deteriorated, and the number decreased, but now CN mitigated the situation (Figure 2(a)). The cell viability of *H. pylori* infection decreased significantly, and it was increased by CN treatment in a concentration-dependent manner (Figure 2(b)). The release of LDH presented the damage of *H. pylori* infection on cells; the release of LDH in *H. pylori*-infected cells increased significantly, and the release of LDH in the CN group decreased gradually with the increase of concentration (Figure 2(c)).

### 3.3. CN Reduced the Apoptosis of H. Pylori-Infected GES-1 Cells

**Figure 3:**
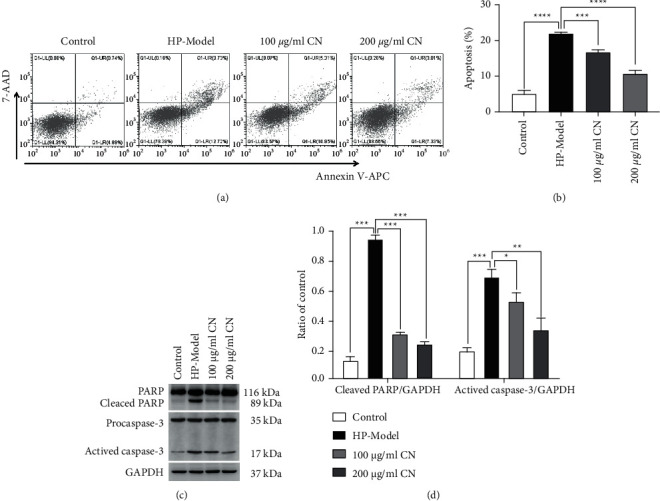
CN reduced the apoptosis of *H. pylori*-infected GES-1. (a, b) Apoptosis detected by flow cytometry. (c, d) Western blotting results of PARP and caspase-3 proteins. ^*∗*^*p* < 0.05, ^*∗∗*^*p* < 0.01, ^*∗∗∗*^*p* < 0.001, compared with the specified group.

The apoptosis of *H*. *pylori*-infected GES-1 cells with or without CN was evaluated by Annexin V/PI double staining and flow cytometry. Data showed that *H. pylori* infection caused 21.73% apoptosis in GSE-1 cells. However, CN treatment prevented GSE-1 cells from apoptosis in a concentration-dependent manner (Figures 3(a) and 3(b)). In order to further clarify the antiapoptotic mechanism, apoptosis-related proteins were detected by Western blotting. Results showed that *H. pylori* infection accumulated the cleaved PARP (89 kDa) and activated caspase-3 (17 kDa), which were both considered to be apoptotic markers. After CN treatment, cleaved PARP (89 kDa) and activated caspase-3 were decreased in a concentration-dependent manner (Figures 3(c) and 3(d)).

### 3.4. CN Decreased ROS Production of *H. Pylori*-Induced GES-1 Cells

**Figure 4:**
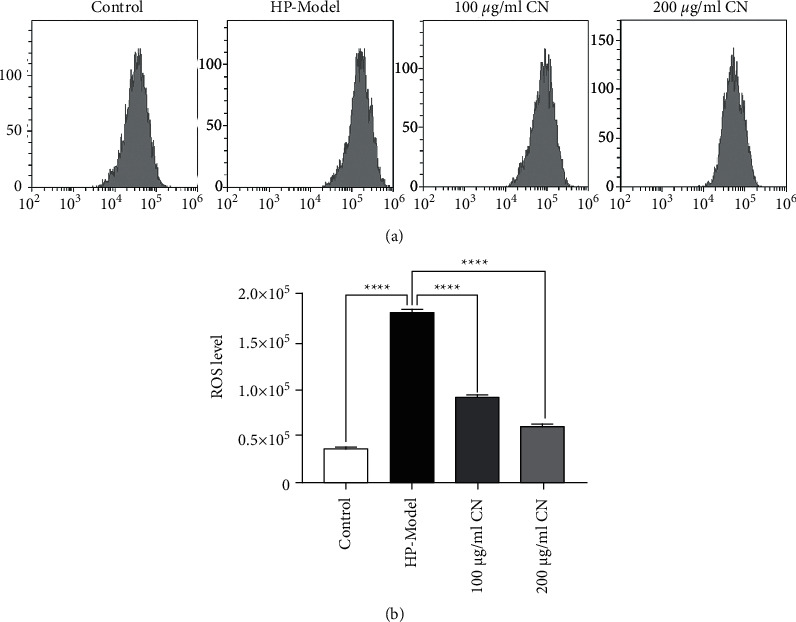
CN decreased the ROS production of *H. pylori*-induced GES-1. (a) ROS production detected by flow cytometry. (b) Quantitative analysis of DCFH-DA accumulation by FACS.

The DCF-DA fluorescence assay was used to evaluate intracellular ROS levels. *H. pylori*-infected GES-1 cells significantly increased the levels of intracellular ROS, and CN reduced ROS levels in *H. pylori*-stimulated GES-1 cells in a dose-dependent manner (Figures 4(a) and 4(b)).

### 3.5. CN Alleviated the Inflammatory Response of *H. Pylori*-Infected Cells

**Figure 5:**
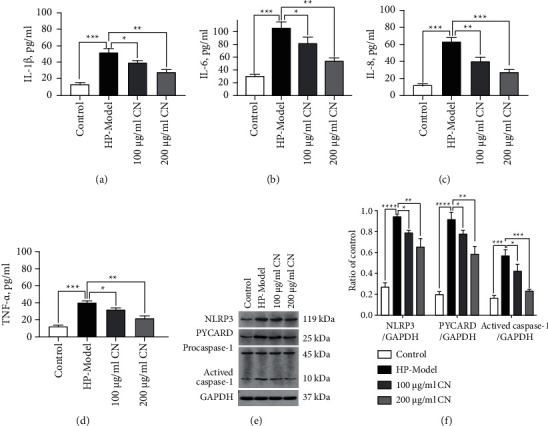
CN alleviated inflammatory response of *H. pylori*-infected GES-1. (a-d) Cellular cytokine productions detected by ELISA. (e-f) Western blotting results of NLRP3, PYCARD, and caspase-1 proteins. ^*∗*^*p* < 0.05, ^*∗∗*^*p* < 0.01, ^*∗∗∗*^*p* < 0.001, compared with the specified group.

Figures 5(a)–5(d) show the effects of CN on the production of cytokines (IL-1*β*, IL-6, IL-8, and TNF-*α*) in *H*. *pylori*-infected GES-1 cells. Cytokines were raised sharply by *H. pylori* infection, and the production of cytokines was significantly reduced by treatment with 100 and 200 *µ*g/ml CN. Besides, *H. pylori* stimulation upregulated the inflammatory protein levels of NLRP3, cleaved caspase-1 (10 kDa), and PYCARD (*p* < 0.001). Interestingly, CN decreased the levels of *H. pylori*-stimulated NLRP3, cleaved caspase-1 (10 kDa), and PYCARD (Figures 5(e)(e) and 5(f)) (*p* < 0.05).

## 4. Discussion

*H. pylori* infection destructs the gastric mucosa of patients and sometimes leads to gastric cancer. There is still great potential to develop drugs to treat *H. pylori* infection given the emergence of side effects and drug resistance. The strategy of natural products paves a promising way for the treatment of *H. pylori* infection-related diseases to effectively clear *H. pylori* with a good effect on improving symptoms of patients. This research found that CN as a natural product showed anti-*H. pylori* activity *in vitro*. More interestingly, our next study showed that the effect of CN on GES-1 cells after *H. pylori* infection was better than that before *H. pylori* infection, suggesting that CN has the potential for the treatment of *H. pylori* infection-related diseases.

The pathogenicity of *H. pylori* is closely associated with numerous virulence factors, including various enzymes, vacuolating cytotoxin, and CagA [21]. Of these, the lipase can destroy the membrane by degrading the membrane lipid skeleton of the *H. pylori*-infected cell, ultimately leading to cell death. The release of cellular LDH into the cell culture medium is regarded as an important indicator of cell membrane integrity. Our data showed that LDH release increased in *H. pylori*-infected GES-1 cells and CN decreased cellular LDH leakage with the increase in concentration. It is worth mentioning that cell viability is not significantly changed when treating GES-1 cells with CN after *H. pylori* infection, which shows that CN may play a certain role in protecting cell integrity and maintaining cell viability. Moreover, our research showed that CN reversed the apoptosis of GSE-1 cells caused by *H. pylori* in a concentration-dependent way, indicating that CN can protect cells from apoptosis during the bacteria infection. At this point, CN can be developed as a valuable clinical ancillary drug to withstand the side effects of front-line chemical drugs in the future.

When *H. pylori* infects the human gastric epithelium, the immune system initiates an immune response to eradicate it. However, *H. pylori* can somehow escape from the capture and elimination of its host immune system. The abnormal free radical metabolism triggered by *H. pylori* can induce lipid peroxidation and produce excessive ROS in tissue cells, ultimately leading to oxidative stress damage to infected cells [22]. In most cases, ROS affects cell signal transduction and causes gastric cancer. Therefore, eliminating ROS levels in *H. pylori*-infected cells may prevent worsening the disease [23]. Our functional experiments revealed that CN reduced the production of ROS in GSE-1 cells caused by *H. pylori* infection in a concentration-dependent manner. The results suggested that CN possessed a certain antioxidant capacity, protecting infected cells from destruction. Numerous studies have proved that the NLRP3 inflammasome can be activated by ROS [24, 25]. Once activated, NLRP3 forms an inflammasome by recruiting PYCARD, where PYCARD interacts with procaspase-1 and transforms it into active caspase-1, converting the cytokine precursors pro-IL-18 and pro-IL-1*β* into the mature IL-18 and IL-1*β*, thus leading to pyroptosis [26]. The results showed that CN could significantly reduce the production of IL-1*β* and other inflammatory factors in *H. pylori*-infected GES-1 cells, and CN effectively reduced the expression of NLRP3, PYCARD, and caspase-1 in *H. pylori*-infected GES-1 cells. The above results suggest that CN protected *H. pylori*-infected GES-1 against apoptosis, which may be related to the inhibition of cellular inflammation. Mechanically, CN may exert anti-inflammatory effects by inhibiting the ROS/NLRP3/caspase-1/IL-1*β* signaling axis [27]. All the evidence suggested that CN had an anti-inflammatory effect in the gastritis cell model related to *H. pylori*. Thus, it should be noted that the antimicrobial capability of CN on probiotics was not detected in this study. A more comprehensive and in-depth study on CN should be considered, including the inflammatory signaling of CN effects and *H. pylori* infection-associated gastritis in vivo. In this way, more research can be conducted on how CN fights *H. pylori* and treats *H. pylori* infection-associated gastritis.

In conclusion, the findings of this study show the antibacterial and anti-inflammatory effects of CN. It can be seen from the results that CN exerted anti-*H. pylori* activity and protected *H. pylori*-infected GES-1 cells by inhibiting ROS/NLRP3/caspase-1/IL-1*β* inflammatory signaling. The above evidence indicates that CN could be considered a promising candidate drug for *H. pylori* infection-associated disease in the future.

## Data Availability

The experimental data used for supporting the results of this study are included in this paper.

## Supplementary Materials

Supplementary Materials Supplementary Figure 1: the plant specimen picture of *Callicarpa nudiflora*. Supplementary Figure 2: the UPLC spectrum of four main compounds. Supplementary Table 1: identification of chemical constituents of *Callicarpa nudiflora* by UPLC-ESI-Q-TOF-MS.
