# Supplementary material for: The Anti-Inflammatory Effect of Callicarpa nudiflora Extract on H. Pylori-Infected GES-1 Cells through the Inhibition of ROS/NLRP3/Caspase-1/IL-1β Signaling Axis
Source: Can J Infect Dis Med Microbiol. 2022 Jul 15;2022:5469236. doi: 10.1155/2022/5469236 (PMC9307406; doi:10.1155/2022/5469236)
Supplement: Supplementary Materials — Supplementary Figure 1: the plant specimen picture of Callicarpa nudiflora. Supplementary Figure 2: the UPLC spectrum of four main compounds. Supplementary Table 1: identification of chemical constituents of Callicarpa nudiflora by UPLC-ESI-Q-TOF-MS. [file 5469236.f1.pdf]

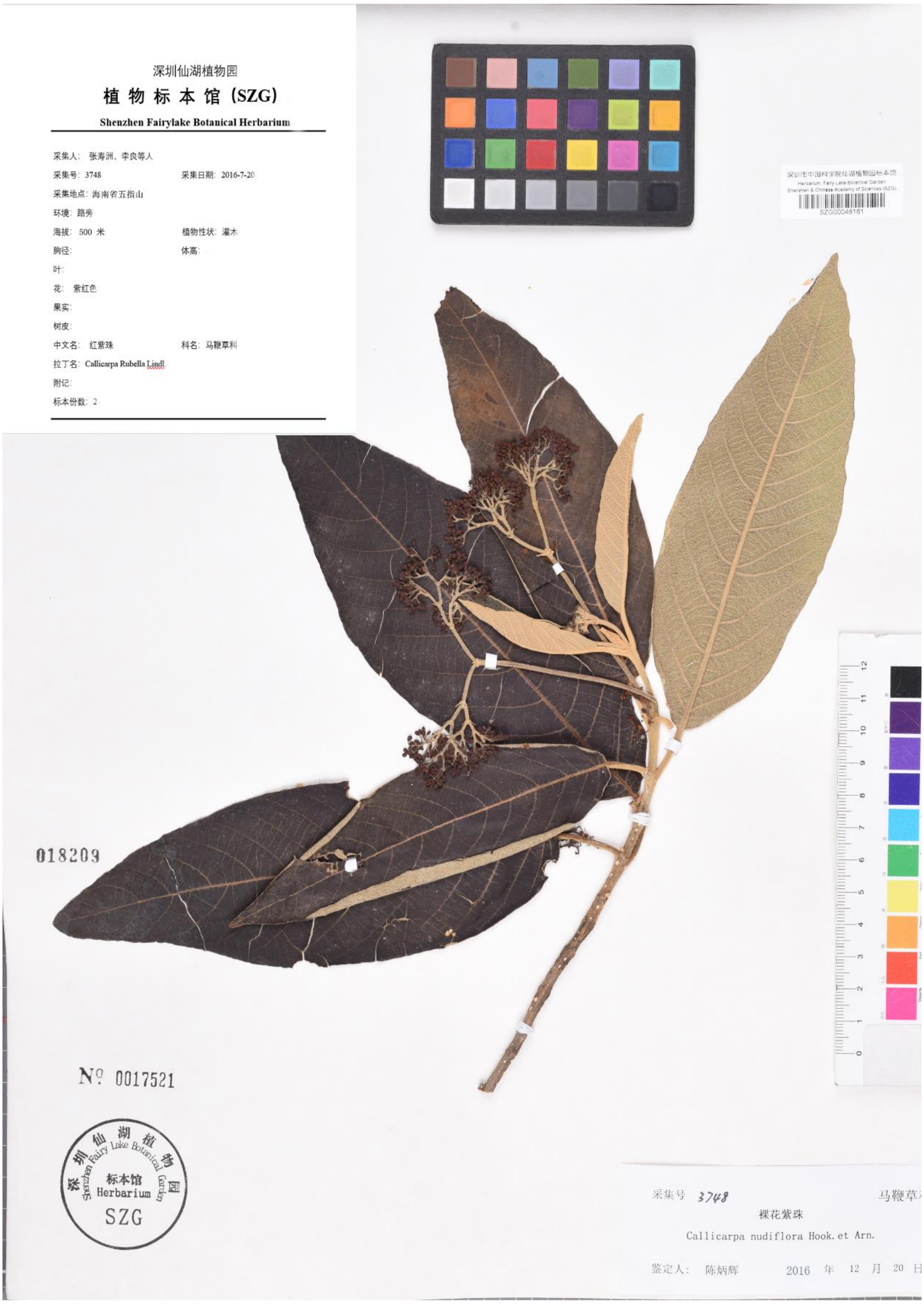

Supplementary Figure 1 The plant specimen picture of *Callicarpa nudiflora*.

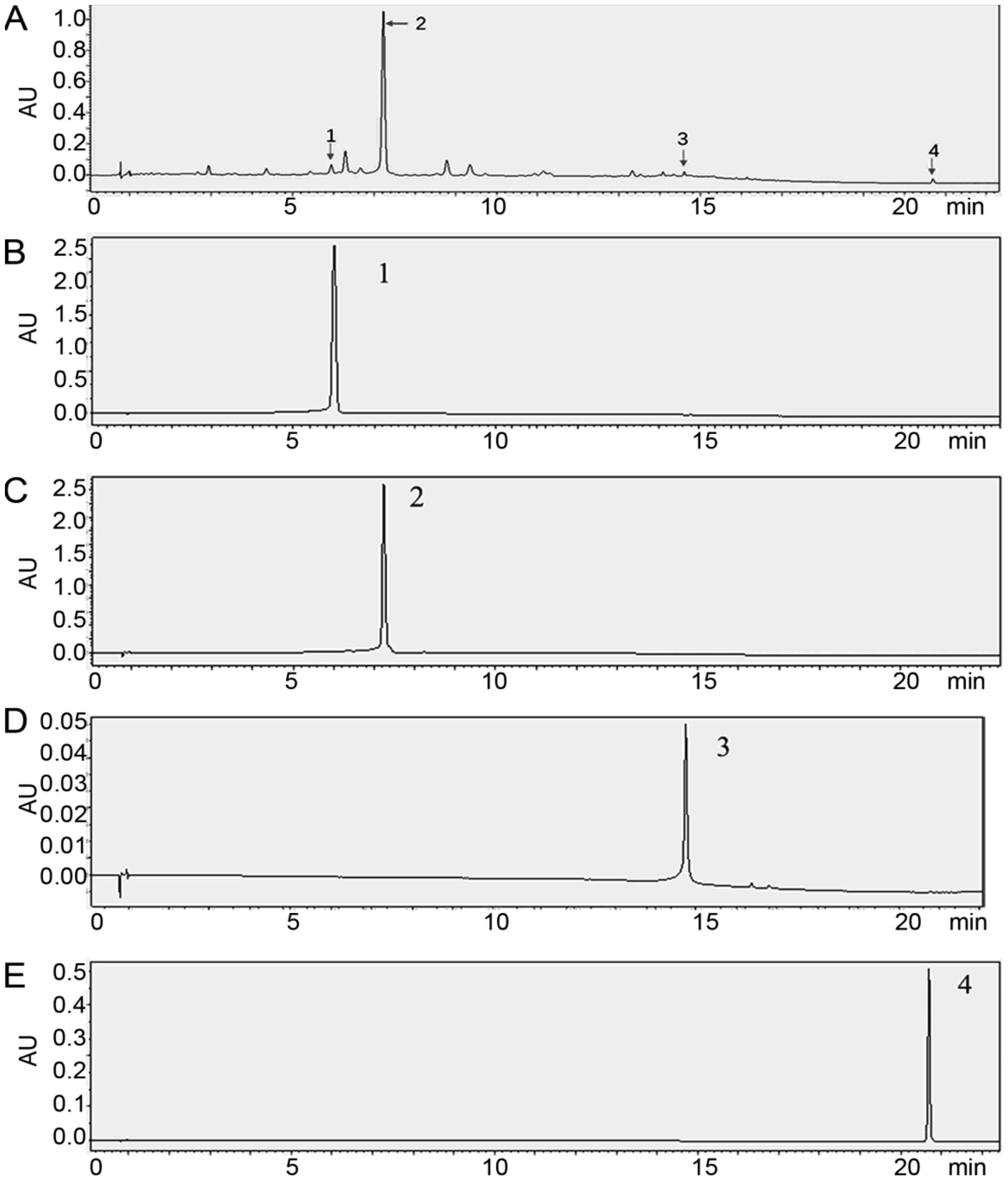

Supplementary Figure 2 The UPLC spectrum of four main compounds.

(A) 1: Forsythoside B (0.98%), 2: Acteoside (33.26%), 3: Luteolin (0.33%),

4: 5,4'-dihydroxy-3,7,3'-trimethoxyflavone (0.42%).

(B-E) The UPLC spectrum of Forsythoside B. (B) Acteoside, (C) Luteolin,

(D) 4: 5,4'-dihydroxy-3,7,3'-trimethoxyflavone, (E) standard sample.

Supplementary Table 1 Identification of chemical constituents of *Callicarpa nudiflora* by UPLC-ESI-Q-TOF-MS

No.	T_R (min)	Identification	Negative ion (m/z)		Positive ion (m/z)		Molecular formula	Molecular weight (Da)	MS/MS (m/z)	Chemical type
			Indicated	ppm	Indicated	ppm				
1	2.05*	catalpol	397.0904	-0.7	-	-	$C_{15}H_{22}O_{10}$	362.1213	-	I
2	5.16	caffeic acid	179.0347	-1.7	-	-	$C_9H_8O_4$	180.0423	-	PA
3	5.35	8-acetyl-harpagide	405.1399	-0.9	429.1356	-1.2	$C_{17}H_{26}O_{11}$	406.1475	(-)165.0552<br>(+)149.0578	I
4	5.82	6′-*O*-*trans*-caffeoylcatalpol	523.1456	-0.2	547.1423	0.1	$C_{24}H_{28}O_{13}$	524.1530	(-)323.0765,161.0241<br>(+)325.0899,163.0369,135.0416	I
5	6.06*	verminoside	523.1456	-0.3	547.1399	-2.3	$C_{24}H_{28}O_{13}$	524.1530	(-)323.0766,179.0345,161.0242<br>(+)163.0380	I
6	6.56	β-hydroxyacteoside	639.1930	-0.1	663.1890	-0.5	$C_{24}H_{28}O_{13}$	524.1530	(-)475.1400,179.0347,161.0244,151.0400<br>(+)163.0383, 135.0421	P
7	6.67	β-hydroxyacteoside	639.1929	-0.2	663.1893	-0.2	$C_{29}H_{36}O_{16}$	640.2003	(-)529.1556,475.1447,179.0347,161.0243,133.0291<br>(+)163.0385,135.0438	P
8	7.18*	6-Hydroxyluteolin-7-O-β-D-glucoside	463.0877	-1.0	465.1013	-1.5	$C_{21}H_{20}O_{12}$	464.0955	(-)301.0346<br>(+)303.0492, 301.0330	F
9	7.88	6-O-*trans*-feruloylcatalpol	537.1604	-1.7	561.1559	-2.0	$C_{25}H_{30}O_{13}$	538.1686	(-)285.0391, 175.0394<br>(+)449.1033,287.0542,177.0543,163.0382,133.0855	I
10	8.04*	forsythoside B	755.2402	-0.3	779.2338	-3.1	$C_{34}H_{44}O_{19}$	756.2477	(-)593.2084,179.0344,161.0242,135.0446<br>(+)163.0383,135.0449	P
11	8.37*	nudifloside	523.1450	-1.3	547.1415	-0.8	$C_{24}H_{28}O_{13}$	524.1530	(-)361.0918,161.0240,133.0290	I

									(+)363.1072,163.0383,135.0427	
12	8.60*	luteoloside	447.0928	-1.1	449.1070	-0.9	$C_{21}H_{20}O_{11}$	448.1006	(-)285.0397<br>(+)287.0548,153.0166	F
13	8.73*	lutedin-7-*O*-neohesperidoside	593.1516	0.3	595.1652	-0.6	$C_{27}H_{30}O_{15}$	594.1585	(-)285.0395<br>(+)287.0541	F
14	8.77*	acteoside	623.1979	-0.3	647.1945	-0.1	$C_{29}H_{36}O_{15}$	624.2054	(-)461.1660,315.1079,179.0346,161.0243,113.0242<br>(+)163.0388,135.0436	P
15	9.52	isoacteoside	623.1976	-0.9	647.1939	-0.7	$C_{29}H_{36}O_{15}$	624.2054	(-)461.1656,315.1070,179.0345,161.0241,135.0446<br>(+)163.0384,135.0430	P
16	9.70	parvifloroside B	623.1978	-0.5	647.1935	-1.2	$C_{29}H_{36}O_{15}$	624.2054	(-)461.1651,179.0345,161.0241,135.0444,133.0289<br>(+)163.0375,135.0419	P
17	9.96*	lutedin-4'-*O*-*β*-*D*-glucoside	447.0928	-1.1	449.1063	-1.6	$C_{21}H_{20}O_{11}$	448.1006	(-)285.0400, 133.0288<br>(+)287.0541	F
18	10.42	chrysoeriol-7-O-β-D-glucoside	461.1082	-1.6	463.1215	-2.0	$C_{22}H_{22}O_{11}$	462.1162	(-)255.0293<br>(+)301.0687	F
19	10.51	10-O-*trans*-p-coumaroylgeniposidic acid	519.1506	-0.4	-	-	$C_{25}H_{28}O_{12}$	520.1581	(-)339.0867,313.1072,163.0395,161.0238	I
20	10.62	luteolin-3'-*O*-β-D-glucopyranoside	447.0932	-0.2	449.1066	-1.2	$C_{21}H_{20}O_{11}$	448.1006	(-)285.0401, 133.0291<br>(+)287.0544,153.0166	F
21	11.22	6'-O-trans-p-coumaroyl-8-epiloganic acid	521.1656	-1.5	545.1618	-1.2	$C_{25}H_{30}O_{12}$	522.1737	(-)297.1125, 195.0654,163.0396<br>(+)179.0689,165.0536	I
22	11.68	martynoside	651.2291	-0.5	675.2249	-1.0	$C_{31}H_{40}O_{15}$	652.2367	(-)175.0396,161.0238,160.0161<br>(+)195.0632,177.0543,163.0736	P

23	12.06*	luteolin	285.0401	-1.3	287.0549	-0.4	$C_{15}H_{10}O_6$	286.0477	(-)179.0344,133.0291<br>(+)339.1055	F
24	14.02*	5,4'-dihydroxy-3,7,3'-trimethoxyflavone	343.0817	-1.9	345.0962	-0.7	$C_{18}H_{16}O_7$	344.0896	(-)328.0577,313.0347<br>(+)330.0726,315.0487	F

*: standard compounds, I: iridoid glycosides, F: flavanones, P: phenylethanoid glycoside, PA: phenylpropionic acid
